# A new species of genus *Rhynchina* Guenée, 1854 from Mt Taibai, China (Lepidoptera, Erebidae, Hypeninae)

**DOI:** 10.3897/zookeys.811.27386

**Published:** 2018-12-31

**Authors:** Hong Zheng, Hui-Lin Han

**Affiliations:** 1 School of Forestry, Northeast Forestry University, Harbin, China Northeast Forestry University Harbin China

**Keywords:** China, Erebidae, Hypeninae, Lepidoptera, new species, *
Rhynchina
*

## Abstract

A new species, *Rhynchinataibaishana* Han, **sp. n.** is described from Mt Taibai, China. The new species is illustrated with images of adults and genitalia, and compared with *R.deqinensis* Han, 2008, *R.helga* Gaal, 1998 and *R.mandarinalis* Leech, 1900.

## Introduction

The genus *Rhynchina* Guenée, 1854 is highly diverse and mostly distributed in the Eastern Palaearctic and Oriental regions. It contains more than 56 described species worldwide (Poole 1989; Lödl 1994, 1997, 1998a, b, c, 1999a, b, 2000; Mayerl and Lödl 1997, 1999; Gaal 1998; Lödl and Gaal 1998; Mayerl 1998; Chen 1999; Han 2008; Hacker et al. 2011; Hacker 2013; Pan and Han 2015; Pekarsky 2017). Among them, 20 species are recorded from China (Chen 1999; Han 2008; Pan and Han 2015; Pekarsky 2017).

In the present study, a new species is described from Mt Taibai, Shaanxi province, China. This new species is compared with its closest relatives, *R.deqinensis* Han, 2008 and *R.helga* Gaal, 1998; some specimens of *R.taibaishana* sp. n. and *R.mandarinalis* Leech, 1900 show also some resemblance in external appearance. All of these species are easily distinguished on the basis of their forewing patterns and configuration of genitalia.

## Materials and methods

All material studied of the new taxon was collected by light trap. Abdomens were macerated in 10% NaOH solution to digest internal tissues; after careful cleaning and removal of scales and contents of coelom, genitalia were examined, compared, and described before being mounted onto microscope slides. Photographs of the adults were taken with a Nikon D300 digital camera and the genitalia were photographed via the Qcapture pro system. Figures were compiled in Adobe Photoshop v. 6.0. The type materials of the new species are deposited in the School of Forestry, Northeast Forestry University, Harbin, China (**NEFU)**.

## Taxonomic account

### Genus *Rhynchina* Guenée, 1854

*Rhynchina* Guenée, 1854, in Boisduval & Guenée, Histoire Naturelle des Insectes, Species Général des Lépidoptères 8: 20. Type species: *Rhynchinapionealis* Guenée, 1854 [Central India].

*Plumipalpia* Hampson, 1898, Journal of the Bombay Natural History Society 11(4): 705. Type species: *Plumipalpialignicolor* Hampson, 1898 [NW Himalayas, Kasauli].

*Rhabinogana* Draudt, 1950, Mitteilungen der münchner entomologischen Gesellschaft 40: 117. Type species: *Rhabinoganaalbistriga* Draudt, 1950 [China, Yangtse Valley, Batang; A-tun-tse].

#### Rhynchina taibaishana

Taxon classificationAnimaliaLepidopteraNoctuidae

Han sp. n.

http://zoobank.org/A742B8FD-9FE7-492E-83B5-8896DF89298A

**Figures 1–6.**
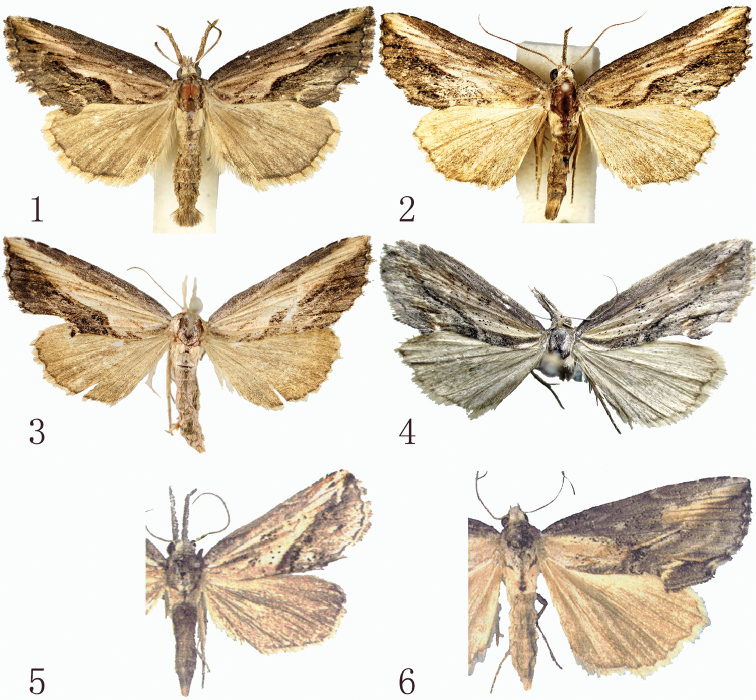
*Rhynchina* spp., adults **1***R.taibaishana* sp. n., male, holotype **2** ditto, male, paratype **3** ditto, female, paratype **4***R.deqinensis* Han, 2008, male, holotype **5***R.helga* Gaal, 1998 (after Mayerl and Lödl 1999) **6***R.mandarinalis* Leech, 1900 (after Mayerl and Lödl 1999).

**Figures 7–10.**
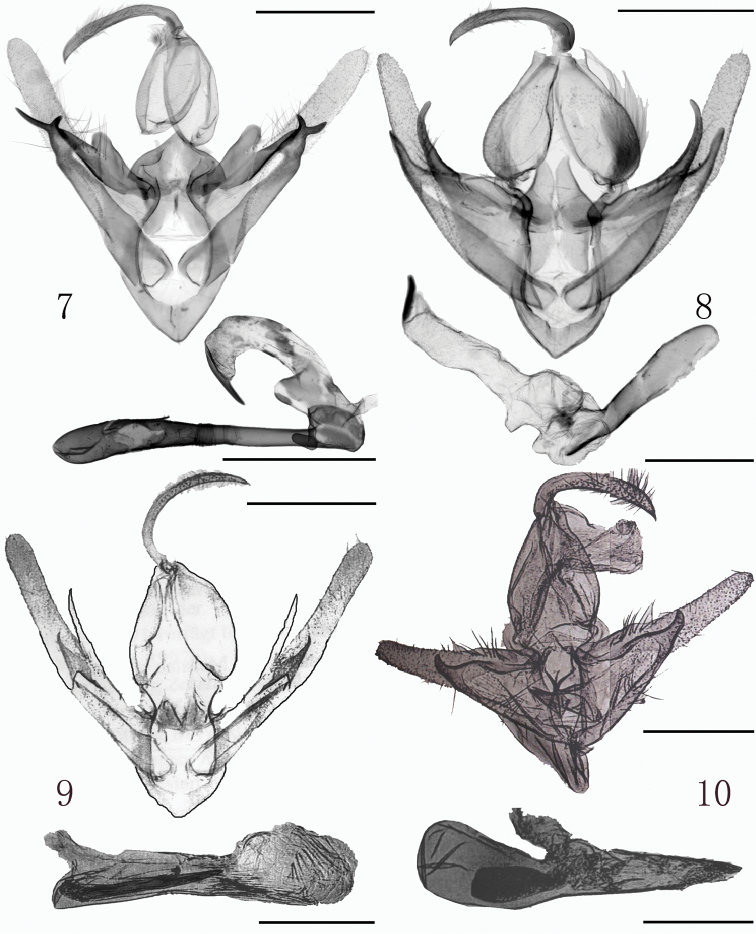
*Rhynchina* spp., male genitalia **7***R.taibaishana* sp. n., holotype **8***R.deqinensis* Han, 2008, holotype **9***R.helga* Gaal, 1998 (after Mayerl and Lödl 1999) **10***R.mandarinalis* Leech, 1900 (after Mayerl and Lödl 1999). Scale bar: 1 mm.

**Figures 11–13.**
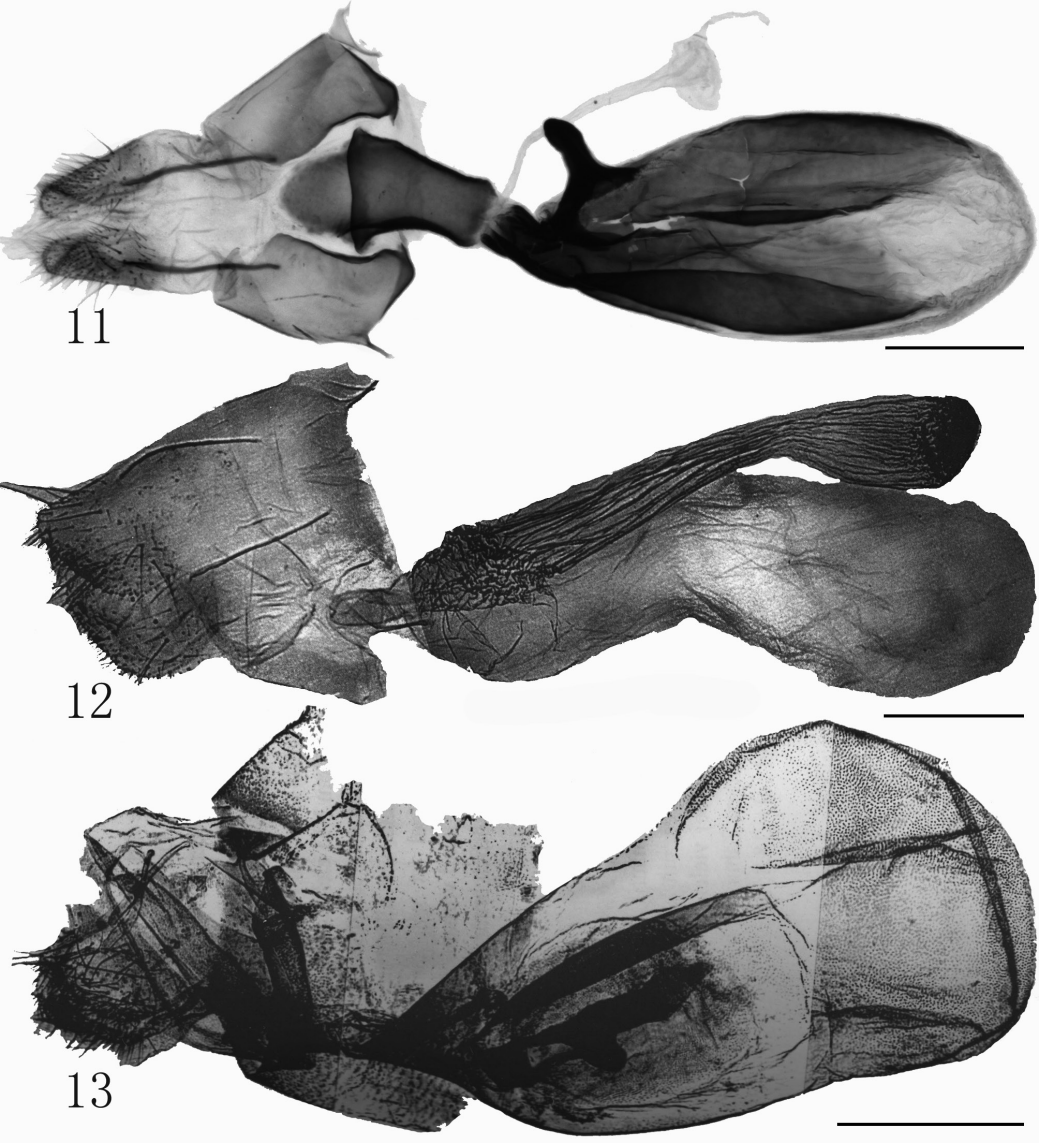
*Rhynchina* spp., female genitalia **11***R.taibaishana* sp. n., paratype **12***R.helga* Gaal, 1998 (after Mayerl and Lödl 1999) **13***R.mandarinalis* Leech, 1900 (after Mayerl and Lödl 1999). Scale bar: 1 mm.

**Figures 14, 15.**
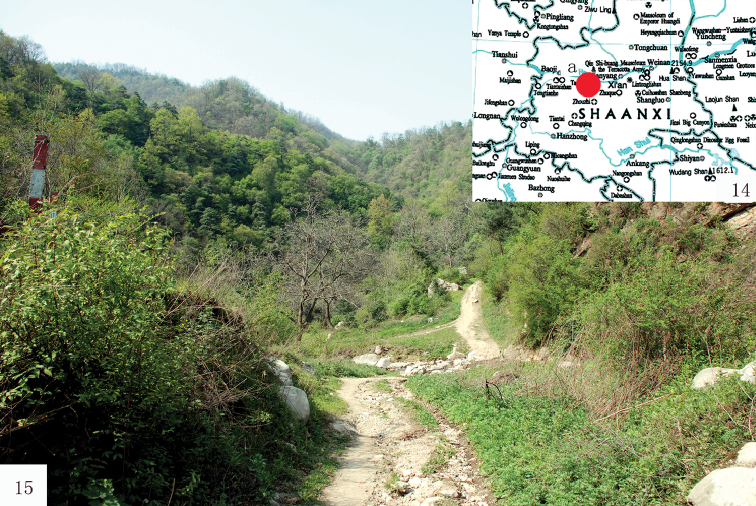
**14** Collection site of *R.taibaishana* sp. n., Haoping protection station **15** Collection site composed of mainly broad-leaved forest and mixed shrubs.

Figures 1–3 , 7 , 11 , 14 , 15

##### Holotype.

♂, China, Shaanxi Province, Mt Taibai, Haoping, 2–10.V.2010, leg. TY. Shao, XW. Liu [NEFU], genit. prep. hhl-2125-1.

##### Paratypes.

1♂, 1♀, same data as holotype [NEFU], genit. prep. hhl-2124-1 (♂), hhl-3817-2 (♀).

##### Diagnosis.

The adult of the new species is similar to *R.deqinensis* Han, 2008 (Fig. 4) and *R.helga* Gaal, 1998 (Fig. 5), but the forewing apex of *R.taibaishana* is sharper than that in *R.deqinensis* and *R.helga*. The postmedial line of *R.taibaishana* undulates more obviously, and bends strongly at CuA_2_, but that of *R.deqinensis* and *R.helga* is smooth. The terminal line is strongly serrated in *R.taibaishana*, but in *R.deqinensis* and *R.helga* it is rather smooth. The orbicular spot of *R.taibaishana* is small, black and indistinct in some specimens, while in *R.deqinensis* and *R.helga* it consists of fine black speckles. In the male genitalia, the costal process of *R.taibaishana* (Fig. 7) is stout and extends over 1/3 the length of valva, but in *R.deqinensis* (Fig. 8) and *R.helga* (Fig. 9) the costal process is very short or small and indistinct. The claspers of *R.taibaishana* are asymmetrical and finger-like, the left one twice as long as the right one, but in *R.deqinensis* they are symmetrical, curved and finger-like, and in *R.helga*, also symmetrical but spine-like. The ampulla in *R.taibaishanna* is short and slightly curved, reaching to the costal margin in the right valva, while that of left valva is somewhat shorter; in *R.deqinensis* and *R.helga*, the ampulla extends along the main axis of valva, while that of *R.helga* is sharp apically and slightly curved. The cornutus of *R.taibaishana* is shorter than that of *R.helga*, and longer than in *R.deqinensis*. In the female genitalia, the corpus bursae of *R.taibaishana* (Fig. 11) is long, oval shaped, its posterior 3/4 sclerotized and bearing a strongly extended sclerotized signum, but the corpus bursae of *R.helga* (Fig. 12) is longer, slightly constricted and bent at the middle and membranous throughout and without a signum, but with large ridged appendix bursae, which is absent in *R.taibaishana*.

Externally *R.taibaishana* is also similar to *R.mandarinalis* Leech, 1900 (Fig. 6), but it differs by the continuous yellow-brown oblique band runing from the apex to the basal part of forewing. In *R.mandarinalis* the forewing shows apical and basal yellowish brown patches, which are connected by a thin, yellowish brown-bordered blackish line. In the male genitalia, the valva of *R.taibaishan* shows parallel costal and ventral margin up to the cucullus, while the valva of *R.mandarinalis* (Fig. 10) is apically tapered. The costa of *R.taibaishan* is strongly developed, while in *R.mandarinalis* it is not expressed. These two species are especially different in the female genitalia (Figs 11, 13), particurlarly in the shape of corpus bursae, which is long-ovoid and sclerotized in the posterior 3/4 in *R.taibaishana*, but broader and pear-shaped, membranous, and bearing small surface granulation in *R.mandarinalis* (Fig. 13). Both species have a strongly sclerotized, outwardly extended finger-like signum on posterior part.

##### Description.

Adult (Figs 1–3). Wingspan 26–29 mm. Head, thorax and abdomen pale yellowish brown with grey scales. Male antenna ciliate. Labial palpi long, upcurved. Forewing yellowish brown, with dark brown and some black suffusion; basal line dark brown, short, arched, feebly distinct; antemedial line black, strongly waved at veins 1A+2A, and distinct only at costal and inner margins; postmedial line double, black, its outer border indistinct at anterior 1/2 and distinct at posterior 1/2 inner border well distinct on costal area, then greatly outwardly produced beyond discal cell, albeit fading in correspondence to pale oblique band bisecting apical area, then slightly undulated and internally oblique to inner margin; subterminal line yellow, a jagged wave, distinct from M_1_ to inner margin, with sharp angle between CuA_2_ and 1A+2A; pale yellowish brown oblique band crosses wing from apex to base; orbicular spot small, dark brown, indistinct; reniform spot dark brown, obscure; tornus extended out with tuft of grayish brown scales; interspaces M_1_-M_2_, M_2_-M_3_ and M_3_-CuA_1_ crossed with a black streak each; costal, adterminal and tornal fields blackish grey; terminal line black; fringe chequered yellowish brown and smoky black, with paler basal dots between the veins. Hindwing light yellowish brown, irrorated with dark brown scales; terminal line thin, black; fringe yellow and black.

##### Male genitalia.

(Fig. 7) Tegumen broad, oblong, 4/5 as long as vinculum. Vinculum V-shaped. Valva narrow, bar-like, elongated; costal process flat, stout, sclerotized and blunt, swollen medially, not reaching middle part of valva; sacculus rather swollen, sclerotized; clasper and ampulla fused, heavily sclerotized, asymmetrical; ampulla short and slightly curved, reaching costal margin on right valva, slightly shorter on left valva; left clasper twice as long as right one, narrow, finely pointed, right one stubby. Uncus long and narrow, bent subbasally, sickle shaped, apical part hooked. Juxta inverted funnel-shaped, sclerotized. Aedeagus long, cylindrical, straight, tapered apically, carina broad, sclerotized; vesica membranous, with broad irregular-shaped basal part, small sack-shaped basal diverticulum, and very long, cylindrical medial diverticulum, armed with long thin apical cornutus connected basally to the vesical membrane for half of its length.

##### Female genitalia.

(Fig. 11) Ostium bursae wider than ductus bursae; antrum cylindrical, sclerotized, slightly curved, and constricted proximally, its dorsal part with a liguliform process, about 1/2 as long as remainder of antrum; ductus bursae very short, about 1/2 length of antrum, sclerotized, joined to this by narrow membranous tract; corpus bursae elongated, ovoid, sclerotized posteriorly for 3/4 of its length, bearing in caudal part strong sclerotized, outwardly extended thumb-like signum, with broad horn-shaped base. Apophyses anteriores very short, broad basally; apophyses posteriors relatively long, about 5 times longer than anteriores; papillae anales elongate, broad.

##### Distribution.

(Fig. 14) China (Shaanxi Province: Mt Taibai).

##### Etymology.

The species name “*taibaishana*” refers to the type locality, Mt Taibai.

##### Bionomics.

(Fig. 15) The species was collected in a broad-leaved forest with shrubs. All individuals have been attracted at ultra violet light in May 2010.

## Supplementary Material

XML Treatment for Rhynchina taibaishana

## References

[B1] Chen YX (1999) Lepidoptera, Noctuidae. Fauna Sinica, Insecta Vol. 16. Science Press, Beijing, 1596 pp.

[B2] Gaal S (1998) *Rhynchinahelgae* sp.n., eine neue Hypeninae (Insecta: Lepidoptera: Noctuidae) aus China. Annalen des Naturhistorischen Museums in Wien 100B: 291–295.

[B3] Hacker H (2013) Additions to several revisions of noctuid genera revised from 2001 to 2011, with descriptions of nine new species and three new subspecies from Africa, Arabian Peninsula and Iran, with faunistic notes (Noctuoidea). Esperiana 18: 199–220.

[B4] Hacker HH Hoppe H Lehmann L Stadie D (2011) Neue Noctuidae-Arten aus Südarabien und Ostafrika (Lepidoptera). Esperiana 16: 233–253.

[B5] Han HL (2008) *Rhynchinadeqingensis* sp. nov., a new Hypeninae from China (Lepidoptera, Noctuidae). Tinea 20(3): 155–157.

[B6] Lödl M (1994) Zur Wiederauffindung der Type von *Rhynchinaobliqualis* (Kollar, 1844) [*Hypena*] comb. nov. im Naturhistorischen Museum Wien, nebst Bemerkungen zur Synonymie (Lepidoptera: Noctuidae). Annalen des Naturhistorischen Museums in Wien 96B: 369–372.

[B7] Lödl M (1997) *Rhynchinabarbarae* sp. n., eine neue zentralasiatische Hypeninae (Lepidoptera: Noctuidae). Entomologische Zeitschrift 107(10): 417–422.

[B8] Lödl M (1998a) *Rhynchinaclaudiae* sp. n., eine neue Hypeninae aus China (Lepidoptera: Noctuidae). Quadrifina 1: 103–107.

[B9] Lödl M (1998b) *Rhynchinaheinrichharreri* sp. n., eine neue Hypeninae aus Nepal (Lepidoptera: Noctuidae). Quadrifina 1: 109–113.

[B10] Lödl M (1998c) *Rhynchinasusannae* sp. n., eine neue Hypeninae aus Nepal (Lepidoptera: Noctuidae). Stapfia 55: 295–297.

[B11] Lödl M (1999a) *Rhynchinamartonhreblayi* sp. n., eine bemerkenswerte neue Hypeninae aus Thailand (Insecta: Lepidoptera: Noctuidae). Annalen des Naturhistorischen Museums in Wien 101B: 349–353.

[B12] Lödl M (1999b) *Rhynchinaangustata* Butler, 1889 jüngeres, subjektives synonym von *R.rivuligera* Butler, 1889 und bemerkungen zu interessanten neufunden aus Thailand (Insecta: Lepidoptera: Noctuidae). Annalen des Naturhistorischen Museums in Wien 101B: 355–357.

[B13] Lödl M (2000) *Rhynchinapanczelosi* sp. n., eine neue Hypeninae aus Nepal (Lepidoptera: Noctuidae). Quadrifina 3: 1–5.

[B14] Lödl M Gaal S (1998) *Rhynchinamichaelhaeupli* sp. n., a new Hypeninae from China (Lepidoptera: Noctuidae). Quadrifina 1: 231–236.

[B15] Mayerl B (1998) Drei neue Arten der Gattung *Rhynchina* Guenée, 1854 aus China (Lepidoptera: Noctuidae: Hypeninae). Quadrifina 1: 93–101.

[B16] Mayerl B Lödl M (1997) Checklist aller Arten der Gattungen *Zekelita* Walker, 1863 und *Rhynchina* Guenée, 1854 der paläarktischen und indo-australischen Region (Insecta: Lepidoptera: Noctuidae). Annalen des Naturhistorischen Museums in Wien 99B: 377–386.

[B17] Mayerl B Lödl M (1999) Revision der Gattung *Rhynchina* Guenée, 1854 (Lepidoptera: Noctuidae: Hypeninae). Quadrifina 2: 1–124.

[B18] Pan ZH Han HL (2015) Description of a new species of the genus *Rhynchina* Guenée, 1854 (Lepidoptera, Erebidae, Hypeninae) from Southeastern Tibet, China. Journal of Forestry Research 26(3): 735–738. 10.1007/s11676-015-0090-4

[B19] Pekarsky O (2017) A new species of *Rhynchina* Guenée, 1854 from China (Lepidoptera, Erebidae, Hypeninae). Entomofauna carpathica 29(1): 1–4.

[B20] Poole RW (1989) Lepidopterorum Catalogus (new series), fascicle 118, Noctuidae. E.J. Brill, Leiden, 1314 pp.

